# Comparison of Bacterial Community Structure and Diversity in Traditional Gold Mining Waste Disposal Site and Rice Field by Using a Metabarcoding Approach

**DOI:** 10.1155/2020/1858732

**Published:** 2020-01-07

**Authors:** Fatimawali, Billy Johnson Kepel, Maria Apriliani Gani, Trina Ekawati Tallei

**Affiliations:** ^1^Pharmacy Study Program, Department of Chemistry, Faculty of Mathematics and Natural Sciences, Sam Ratulangi University, Manado, Indonesia; ^2^Department of Chemistry, Faculty of Medicine, Sam Ratulangi University, Manado, Indonesia; ^3^Department of Biology, Faculty of Mathematics and Natural Sciences, Sam Ratulangi University, Manado, Indonesia

## Abstract

Traditional small-scale gold mining mostly use mercury to extract the gold from ores. However, mercury contamination in the environment can affect the composition and structure of the bacterial community. The purpose of this study was to determine the effect of mercury contamination on the bacterial community in the traditional gold mining waste disposal site and in the rice field. Mercury analysis was carried out using the CVAFS method. Analysis of bacterial communities and structure was carried out based on the results of metabarcoding of the V3-V4 16S rRNA regions obtained from paired-end Illumina MiSeq reads. The results showed that the sample from the mining waste disposal site had a mercury level of 230 mg/kg, while the sample from the rice field had 3.98 mg/kg. The results showed that there were differences in microbial composition and community structure in both locations. With the total reads of 57,031, the most dominant phylum was Firmicutes in the mining disposal site sample. Meanwhile, with the total reads of 33,080, the sample from rice field was dominated by Planctomycetes. The abundant classes of bacteria in the mining waste disposal site, from the highest were Bacilli, Gammaproteobacteria and Planctomycetia, while the sample from the rice field was dominated by the Planctomycetia and Acidobacteria subdivision 6. The families that dominated the sample in disposal site were Bacillaceae and Aeromonadaceae, while the sample from the rice field was dominated by Gemmataceae. The abundant genera in both locations were *Bacillus* and *Gemmata*. This study concluded that the high level of mercury in the soil reduced the richness and diversity of bacterial phyla and lower taxa. There was also a shift in the dominance of phyla and lower taxa in both locations. This study provides an understanding of the microbial community structure in the area that is highly contaminated with mercury to open insight into the potential of these bacteria for mercury bioremediation.

## 1. Introduction

Mercury is known as one of the heavy metals that is very toxic in the environment and can affect human and animal health [1, 2]. It exists in nature in three different forms with different toxicity, usage, and properties. The three forms of compounds are organic mercury, inorganic mercury, and elemental or metallic mercury [3]. Despite its toxic properties, mercury is still widely used by the community in North Sulawesi as an ingredient to extract gold from the soil or ore as amalgam [4, 5]. Unfortunately, the waste from this activity is discharged freely without processing beforehand to be more friendly to the environment [2]. If mercury is discharged into the environment, it will be methylated to methylmercury, and its concentration can increase at each level of the food chain. Boese-O'Reilly et al. [6] reported that blood, urine, and hair mercury levels from children (8 to 13 years) who worked as gold miners in Tatelu, North Sulawesi, were much higher than those in the control group. These children showed symptoms of ataxia. Other adverse effects that can arise due to mercury exposure include neurotoxicity, nephrotoxicity, teratogenicity (Minamata disease), increased risk of a heart attack and hypertension, cancer, and gene mutation [7].

Mercury detoxification is one way to reduce mercury pollution, for example, by using mercury-resistant bacteria. Previous studies reported that areas contaminated with mercury were identified to contain mercury-resistant bacteria [2, 4, 5, 8–10]. Mercury-resistant bacteria are bacteria that can mediate the enzymatic reduction process from toxic mercury to volatile mercury [2, 11]. If these bacteria can adapt to environments with high levels of heavy metal contamination, then the use of these bacteria is very effective in increasing the reduction of heavy metals.

Fatimawali et al. [4] succeeded in isolating mercury-resistant *Klebsiella pneumoniae* from mercury-contaminated areas. This bacterium reduced HgCl_2_ 75% in 1 hour, 92% in 12 hours, and 99.4% in 24 hours. *Pseudomonas* sp. that was resistant to mercury had also been successfully isolated from mercury-contaminated environment [5]. This bacterium reduced the phenyl mercury levels in the media to 74.99% within 24 hours of incubation. With this process, mercury-resistant bacteria can be used as bioremediation agents for mercury, even more convincing and potential than other conventional contemporary remediation [11]. For this reason, bacterial communities in mercury-polluted locations need to be studied to understand the diversity of bacteria that have the potential as mercury bioremediation agents. This research was aimed at identifying bacteria living in mercury-contaminated areas and compared them with bacteria in areas that contained a very little amount of mercury. Community structure and bacterial diversity in the places were then compared.

## 2. Materials and Methods

### 2.1. Study Area and Sample Collection

Soil samples were collected from traditional mining sites which used mercury metal to extract gold from ore in North Tanoyan Village, Bolaang Mongondow Regency, North Sulawesi, at an altitude of 2000 feet (500 meters) above sea level. At that location, there are three traditional gold minings which have been operating for more than 10 years. The first sample was taken from the mining waste disposal hole (location A), and the second sample was taken from the rice field (location B) which was about 100 meters from the mine waste disposal site. Sterile polyethylene tubes were used as soil containers. Samples were taken to the laboratory using a cooling box for further analysis of mercury content and bacterial composition, community structure, and diversity.

### 2.2. Measurement of Mercury Levels of the Samples

For mercury content analysis, 0.20 g of each soil was extracted with 10 mL mixed solution (2 mol/L HNO_3_ and 4 mol/L HCl) in a Teflon tube at 95°C for 2 h. The total amount of Hg in these extracts was determined via cold vapor atomic fluorescence spectrometry (CVAFS) (USEPA-3050-B and USEPA 245.7).

### 2.3. DNA Extraction, PCR Amplification, 454 Pyrosequencing, and High-Throughput Sequencing Data Processing

The genomic DNAs (gDNAs) of bacteria were extracted from soil using ZymoBiomics DNA Mini kit (Zymo Research) according to the protocol provided by the manufacturer. Amplification of hypervariable V3-V4 regions of 16S rRNA were performed using MyTaq™ HS Red Mix (Bioline, BIO-25044) in Agilent SureCycler 8800 Thermal Cycler. The reaction conditions were as follows: initial denaturation at 95°C for 3 min, followed by 35 cycles of denaturation at 95°C for 15 sec, annealing at 52°C for 30 sec, extension at 72°C for 45 sec, and then followed by final extension at 72°C for 3 min. Preparation of 16S rRNA libraries and bioinformatic analysis were performed following the previous research [12].

### 2.4. Analysis of Bacterial Diversity

The alpha and global beta diversities of the bacterial gut were calculated and analysed using PAST3 v. 3.24 [13].

## 3. Results and Discussion

### 3.1. Mercury Level of Samples

Mercury concentrations in both soil samples were analysed using CVAFS. The soil sample in location A had a high mercury concentration of 230 mg/kg (230 ppm). The soil sample obtained from location B which located 100 meters from location A had a much lower mercury concentration of 3.98 mg/kg (3.98 ppm). Both locations were separated by highways, but connected by a river. Vishnivetskaya et al. [14] reported that with increasing distance from high levels of mercury-contaminated locations, inorganic mercury levels decreased, while Me-Hg levels increased, indicating mercury is a bioavailable compound and can be accessed by resident microorganisms. Revis et al. [15] suggested that an acceptable limit of soil mercury was 72 ppm. The World Health Organization (WHO) suggested that the provisional tolerable weekly intake (PTWI) of mercury is 1 *μ*g/kg body weight [16]. The mercury in paddy fields may contribute to the level of mercury in rice which needed to be studied further. Feng et al. [17] reported that the main exposure of Me-Hg in human was through the frequent consumption of rice meals. Long-term consumption of mercury-contaminated rice grain may further pose serious health risks.

### 3.2. Bacterial Composition

**Figure 1:**
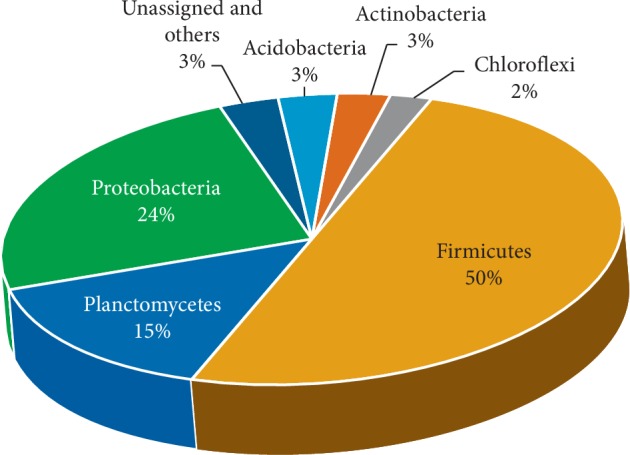
The percentage of bacterial phylum dominance at location A.

**Figure 2:**
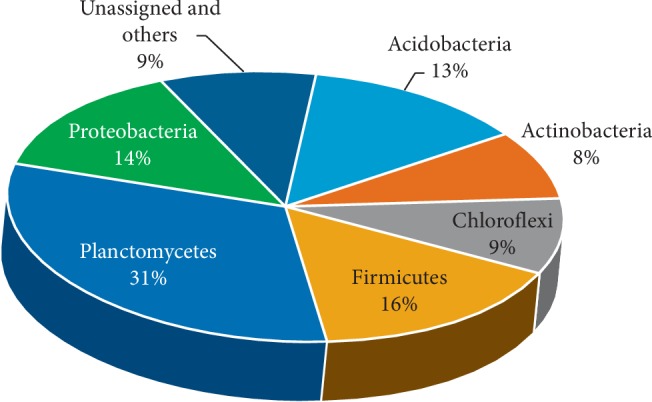
The percentage of bacterial phylum dominance at location B.

Metabarcoding analysis of 16S rRNA V3-V4 regions revealed that there were 57,031 reads (2,694 OTUs) in the sample from location A and 33,080 reads (2,759 OTUs) in the sample from location B, both consisting of 15 phyla of the kingdom bacteria and 2 phyla of the kingdom archaea (Crenarchaeota and Euryarchaeota) with a very limited amount in both locations. Phyla abundances in both locations are presented in Figures 1 and 2. Firmicutes (50%) was the most abundant phylum at location A, followed by Proteobacteria (24%). The soil in location B was dominated by Planctomycetes (31%), followed by Firmicutes (16%) and Proteobacteria (14%). The shift in bacterial dominance from Firmicutes to Planctomycetes was associated with the reduction in mercury concentration. It also may be caused by different physical and chemical factors and soil nutrients in each sample that affected bacterial growth.

Liu et al. [9] reported that mercury concentrations had a positive effect on the abundance of Firmicutes and Bacteriodetes on soils in rice fields and highland. Another study by Rothenberg et al. [18] which used several mercury biomarkers (stool, hair, and cord blood) revealed that Firmicutes were the most abundant phylum (56%). It also has been reported that the presence of mercury alters the bacterial community structure and diversity in soil [19].

This is in line with the current research that Firmicutes was the most abundant phylum in location A, where the mercury content was 57 times higher than in location B. Susilowati et al. [20] reported that Firmicutes was the most abundant phylum in the area around rice fields. Meanwhile, Proteobacteria were also amongst the abundant phyla in location A after Firmicutes. This is in line with the research of Mahbub et al. [19], where Proteobacteria (21.95%) was the abundant phylum in soil samples containing inorganic mercury after Actinobacteria (22.65%). Proteobacteria (14%) was also found in location B. Previous research reported that Proteobacteria was one of the Hg-methylators identified in wastewater [10], and the abundance of this phylum reached 37.8% in the rice fields. Even this phylum was the most abundant in mercury-contaminated rice fields [9].

Unlike the sample at location A, the sample at location B was dominated by Planctomycetes. This phylum was commonly found in agricultural soils [21] and on the banks of lakes [22]. However, this phylum was also identified at location A by 15%. Xu et al. [10] reported that Planctomycetes was identified on soil contaminated with mercury, although it was not the most dominating phylum. In phylum Firmicutes, Bacilli was the most abundant class at location A (45%) and was the second in location B (14%). Several previous studies reported that several genera and even species belonging to this class were found in areas contaminated with mercury, such as in Japan [23], India [24], Mexico [25], Northwestern England [26], Kolyma Lowland and Canada [27], and Indonesia [28]. Chatziefthimiou et al. [29] reported that some bacteria in the order Bacillales were resistant to mercury to a concentration of ±200 *μ*M HgCl_2_, where most isolates were identified as having gen *mer*A.

Gammaproteobacteria (23%.) was the abundant class in location A after Bacilli (22.9%). Møller et al. [8] reported that Gammaproteobacteria were mercury-resistant bacteria that dominated freshwater (56%) and snow (42%) with mercury concentrations in the range 70–80 ng/L. In addition, one of the isolates in the Gammaproteobacteria had the ability to reduce Hg (II) to Hg (0). As many as 76% of the MerA sequences identified in this class had 99-100% amino acid sequences similar to Tn5042 and Tn5041. Planctomycetia (14.6%) was one of the most abundant classes of bacteria at location A and was the third after Bacilli and Gammaproteobacteria. Chen et al. [30] reported that *Pirellula* (class: Bacilli) dominated the Dongdagou river which was known to be high in cadmium, arsenic, lead, and mercury. Acidobacteria subdivision 6 (10%) was abundant in location B. These bacteria had detoxification operon against mercury [31]. On the contrary, Zhang et al. [32] reported that these bacteria were the most abundant class in soil and took up 28.30% of the total reads.

At the family level, location A was dominated by Bacillaceae (28.75%; 16398 reads). One of the genera, *Bacillus*, was found (7.6%; 4355 reads) at location A and also identified in location B (8.56%; 2831 reads). Bacillaceae is a Gram-positive, aerobic or facultative anaerobic bacteria, rod-shaped, and chemoorganotrophic. Several *Bacillus* species were found to have broad spectrum mer operon [33]. This operon encodes for proteins involved in Hg regulation, binding, and organomercury degradation [34] so that it is responsible for bacterial resistance to mercury [35, 36]. TNMERI1 is a class II transposon that has a broad spectrum mercury-resistant genes. Narita et al. [23] reported that 21 of 56 *Bacillus* species isolated from 15 different places in the world had transposons that resembled TNMERI1, which were then classified into Tn5084, Tn5085, and TNMERI1. This may contribute to the horizontal spread of mer operon between *Bacillus* species [23, 26]. Some *Bacillus* isolated in Surabaya, Indonesia, were resistant to mercury at a concentration of 25 mg/L HgCl_2_ [37].

Aeromonadaceae was identified with a large number of location A with 11028 reads. Aeromonadaceae was one of the families found in a gold mining area contaminated with mercury in Bandung, Indonesia [38]. Gemmataceae was the most abundant family in the location of B. *Gemmata* (family: Gemmataceae) was identified as many as 1812 reads at location A and 2393 reads at location B. Previous research reported that this genus was identified on wetlands [39], freshwater, and soil [40]. Previous studies reported that some potential mercury-resistant bacteria were isolated from soil containing high levels of mercury [2, 4, 5, 8–10, 41, 42].

**Figure 3:**
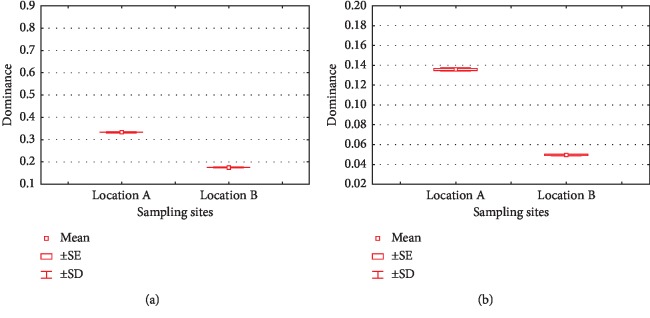
Dominance indices of phylum (a) and lower taxa (b) in location A and B.

**Figure 4:**
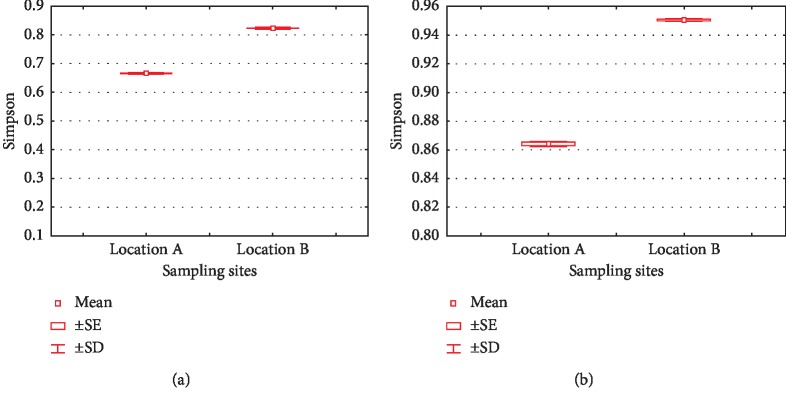
Simpson indices of phylum (a) and lower taxa (b) in location A and B.

**Figure 5:**
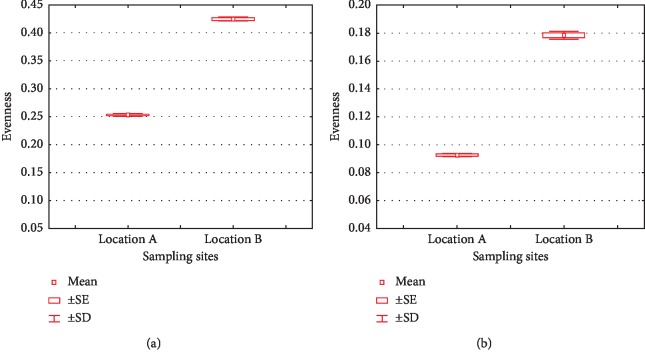
Evenness indices of phylum (a) and lower taxa (b) in location A and B.

**Figure 6:**
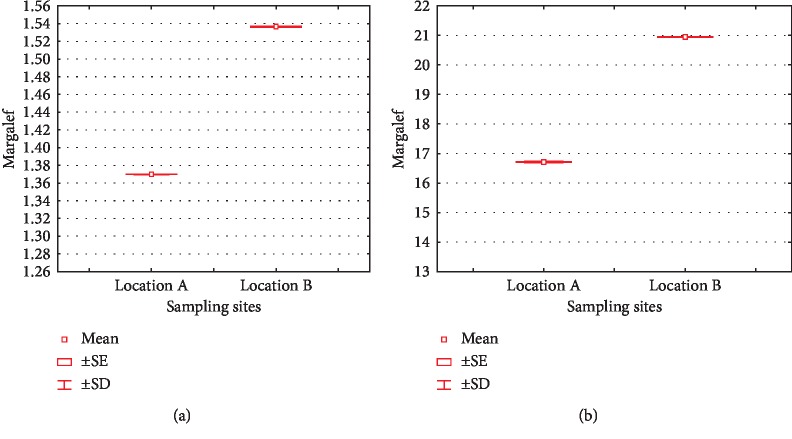
Margalef indices of phylum (a) and lower taxa (b) in location A and B.

**Figure 7:**
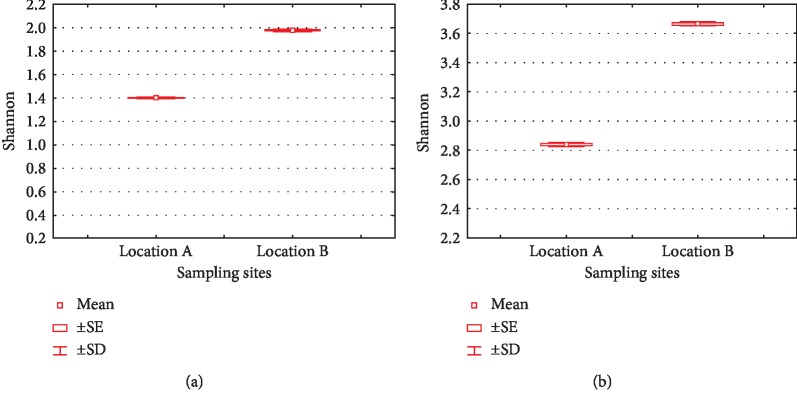
Shannon–Wiener indices of phylum (a) and lower taxa (b) in locations A and B.

**Figure 8:**
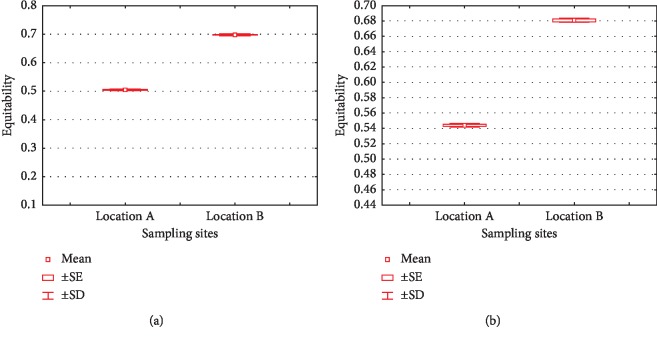
Equitability indices of phylum (a) and lower taxa (b) in location A and B.

Mercury is a dangerous compound that can cause toxic effects, depending on the chemical form and route of its exposure [43]. However, some types of bacteria can be resistant to mercury [8]. For example, *Brevundimonas vesicularis* which was resistant to mercury at concentrations of 10, 20, and 30 ppm [42]. *Klebsiella pneumoniae* and *Pseudomonas* sp. were reported to be highly resistant to mercury [4, 5]. *Pseudomnas plecoglossicida* showed a very high level of tolerance to mercury [44]. Other study reported that *Serratia*, *Streptococcus*, and *Enterococcus* were resistant to mercury to a concentration of 150 ppm [45]. These bacteria have the potential to be developed as bioremediation agents at heavy metal-polluted sites. Alpha diversity of bacterial communities found in locations A and B is represented by dominance, evenness, Margalef, Simpson, Shannon–Wiener, and equitability indices (Figures 3–8). Dominance index (*D*) of both samples are presented in Figure 3. The *D* value of phylum (Figure 3(a)) and lower taxa (Figure 3(b)) in both samples indicated that there were neither phyla nor lower taxa dominated the bacterial community as a whole. The Simpson index (1 – *D*) gives the probability that two individuals taken randomly in an area will belong to the same species/lower taxa [46, 47]. This index focuses more on the dominant species, where rare species will not have a major impact on diversity [47]. This value ranges from 0 to 1, in which 1 indicates a very diverse community, while 0 indicates the absence of diversity. The Simpson index in both locations can be seen in Figure 4. Location B had a value of 0.8247 for phyla and 0.9505 for lower taxa. This indicates that the bacterial community at location B was more diverse than location A. This index takes into account the lower taxa richness and evenness of the abundant lower taxa. The higher the richness and evenness, the lower the dominance, and even the higher the diversity will be [46, 47].

The evenness index (*e*^H^/*S*) describes how similar one species is to another in terms of species abundance. Its value is presented in Figure 5, where high values were seen in location B (0.4251 for phyla). The value is between 0 and 1, while the lower evenness value indicates the more uneven distribution, which makes certain phyla or lower taxa dominate the community [48], and vice versa. The lower value of this in location B for phylum and lower taxa indicated the less evenness in the bacterial community; therefore, some phyla or lower taxa dominated the community. The evenness of lower taxa in location A was also low. A slightly higher evenness index in location A for phyla indicates that there was no dominance in the community.

Margalef index (taxa richness) is the simplest index in biodiversity [46]. This index has the following criteria: if *R* < 2.5 then taxa richness is low, if 2.5 > *R* > 4, then taxa richness is fairly moderate, and *R* > 4 states a high taxa richness. As seen in Figure 5, location B was an area with a high Margalef index value for phylum and lower taxa. High species richness indicated high stability in an ecosystem, thus enabling the ecosystem to be more resistant to natural and anthropogenic disturbances [49].

The value of the Shannon–Wiener Index (*H*′) can be seen in Figure 7. Based on the *H*′ value, the diversity of phyla in both locations was relatively low, while the diversity of lower taxa at location A was moderate (2.836), while it was high (3,665) in location B. As is the case with the Simpson index, this is because lower taxa richness and evenness were high, but dominance was low, making diversity even higher [46, 47].

Equitability index (*J*) is an index developed by Pielou [50], as one of the derivatives of the Shannon index which has a range from 0 (low uniformity) to 1 (high uniformity).  Figure 8 illustrates the *J* values of phyla and lower taxa. It is seen that location B has the highest J value, both in phyla (0.6981) and lower taxa (0.6801). Research of Ji et al. [44] showed similar results that the diversity and richness of bacteria in areas with less pollution would be higher than areas with high pollution. The high level of heavy metals can affect bacterial diversity, population size, and activities [51].

Global beta diversity was calculated using PAST3. Whittaker index for phyla, class, and lower taxa were 0.03, 0.17, and 0.23, respectively. Global beta diversity indicates that the higher beta diversity index means the two communities are more dissimilar. This implies that both sites shared common phyla, class, and lower taxa of bacterial community.

## 4. Conclusions

Both samples contained very different levels of mercury. Samples taken from mine waste disposal site (location A) had a very high mercury level (230 mg/kg) and rice field (location B) had a lower mercury levels (3.98 mg/kg). The most dominating phylum in location A was Firmicutes and in location B was Planctomycetes. Therefore, there was a shift in the dominance of phyla between the two locations. The most abundant class of bacteria in location A was Bacilli, while at location B was Planctomycetia. The most dominating family in location A was Bacillaceae, while in location B was Gemmataceae. The abundant genera in both locations were *Bacillus* and *Gemmata*. This study concludes that high level of mercury in the soil reduced the richness and diversity of the bacterial community.

## Data Availability

The data used to support the findings of this study are available from the corresponding author upon request.
